# Human Microbiota and Breast Cancer—Is There Any Relevant Link?—A Literature Review and New Horizons Toward Personalised Medicine

**DOI:** 10.3389/fmicb.2021.584332

**Published:** 2021-02-25

**Authors:** Diogo Alpuim Costa, José Guilherme Nobre, Marta Vaz Batista, Catarina Ribeiro, Catarina Calle, Alfonso Cortes, Maximilian Marhold, Ida Negreiros, Paula Borralho, Miguel Brito, Javier Cortes, Sofia Azambuja Braga, Luís Costa

**Affiliations:** ^1^Breast Cancer Unit, CUF Oncologia, Lisbon, Portugal; ^2^NOVA Medical School, Faculdade de Ciências Médicas, Lisbon Portugal; ^3^Faculdade de Medicina, Universidade de Lisboa, Lisbon, Portugal; ^4^Medical Oncology Department, Hospital Prof. Doutor Fernando Fonseca, Amadora, Portugal; ^5^Faculdade de Medicina, Universidade de Coimbra, Coimbra, Portugal; ^6^Faculdade de Ciências da Saúde, Universidade da Beira Interior, Covilhã, Portugal; ^7^Pathology Department, CUF Oncologia, Lisbon, Portugal; ^8^Medical Oncology Department, Hospital Universitario Ramón Y Cajal, Madrid, Spain; ^9^Division of Oncology, Department for Medicine I, Medical University of Vienna, Vienna, Austria; ^10^Health and Technology Research Center (H&TRC), Escola Superior de Tecnologia da Saúde de Lisboa, Instituto Politécnico de Lisboa, Lisbon, Portugal; ^11^International Breast Cancer Center (IBCC), Quiron Group, Barcelona, Spain; ^12^Vall d’Hebron Institute of Oncology (VHIO), Barcelona, Spain; ^13^Medica Scientia Innovation Research, Valencia, Spain; ^14^Medical Oncology Department, Hospital de Santa Maria, Centro Hospitalar de Lisboa Norte, Lisbon, Portugal; ^15^Instituto de Medicina Molecular João Lobo Antunes, Faculdade de Medicina, Universidade de Lisboa, Lisbon, Portugal

**Keywords:** breast cancer, microbiota, microbiome, dysbiosis, metabolomics, pharmacomicrobiomics, personalized medicine, review

## Abstract

Breast cancer (BC) is the most common malignancy and the second cause of cancer-specific death in women from high-income countries. Recently, gut microbiota dysbiosis emerged as a key player that may directly and/or indirectly influence development, treatment, and prognosis of BC through diverse biological processes: host cell proliferation and death, immune system function, chronic inflammation, oncogenic signalling, hormonal and detoxification pathways. Gut colonisation occurs during the prenatal period and is later diversified over distinct phases throughout life. In newly diagnosed postmenopausal BC patients, an altered faecal microbiota composition has been observed compared with healthy controls. Particularly, β-glucuronidase bacteria seem to modulate the enterohepatic circulation of oestrogens and their resorption, increasing the risk of hormone-dependent BC. Moreover, active phytoestrogens, short-chain fatty acids, lithocholic acid, and cadaverine have been identified as bacterial metabolites influencing the risk and prognosis of BC. As in gut, links are also being made with local microbiota of tumoural and healthy breast tissues. In breast microbiota, different microbial signatures have been reported, with distinct patterns per stage and biological subtype. Total bacterial DNA load was lower in tumour tissue and advanced-stage BC when compared with healthy tissue and early stage BC, respectively. Hypothetically, these findings reflect local dysbiosis, potentially creating an environment that favours breast tumour carcinogenesis (oncogenic trigger), or the natural selection of microorganisms adapted to a specific microenvironment. In this review, we discuss the origin, composition, and dynamic evolution of human microbiota, the links between gut/breast microbiota and BC, and explore the potential implications of metabolomics and pharmacomicrobiomics that might impact BC development and treatment choices toward a more personalised medicine. Finally, we put in perspective the potential limitations and biases regarding the current microbiota research and provide new horizons for stronger accurate translational and clinical studies that are needed to better elucidate the complex network of interactions between host, microorganisms, and drugs in the field of BC.

## Introduction

Breast cancer (BC) is the most common malignancy and the second cause of cancer-specific death in women from high-income countries. BC incidence and mortality rates vary greatly worldwide. According to global cancer statistics from the International Agency for Research on Cancer (IARC), over 2 million new cases and 600 thousand deaths from BC were documented in 2018 (Ferlay et al., 2018). The 5 year overall survival for BC is 98% for localised disease, 84% for regional disease, but only 23% for distant disease (Saraiva et al., 2017).

The most relevant known BC risk factors include exposure to endogenous and exogenous oestrogens, high breast density, history of atypical hyperplasia, lifestyle factors, such as the Western-style diet, obesity and alcohol consumption, and genetic susceptibility (Cardoso et al., 2019).

Current evidence points to other clues for a complementary mechanism of non-hereditary risk of BC. Infectious agents are known to be the third most important risk factor, after tobacco and obesity, contributing to 15–20% of cancer incidence (Garrett, 2015; Banerjee et al., 2018). Gut microbiota is an emerging field of research, that is being associated with cancer, through direct and indirect interference in diverse biological processes: host cell proliferation and death, immune system function, chronic inflammation, oncogenic signalling, hormonal and detoxification pathways. Better understanding about this nested and complex interplay between the host and its own microbes has provided important data about how gut microbiota plays a crucial role on the development, treatment and prognosis of cancer, in particularly BC (Garrett, 2015; Roy and Trinchieri, 2017; Banerjee et al., 2018; Dart, 2019; Alpuim Costa et al., 2020a). For example, a distinct local and/or intestinal microbiota signature may contribute to BC development and an “infected” tumour microenvironment can provide a special fertile habitat for cancer and its stromal cells.

Most BC patients are diagnosed in initial stages when the goal of treatment is cure. In early and locally advanced BC a multimodal approach is frequently used, incorporating surgery, radiotherapy and systemic therapy (Cardoso et al., 2019). According to real-world and clinical trial data, metastatic BC has a highly variable clinical course, with some patients dying a few months after diagnosis and others surviving more than 10 years. Therefore, the primary goals of treatment are to prolong survival and ameliorate the quality of life (Saraiva et al., 2017; Cardoso et al., 2019). Even with standardised treatment protocols, patients do not respond in similar ways, reflecting the heterogeneity of each individual, their unique BC features, and tumour microenvironment (Cardoso et al., 2019). However, little is still known about why analogous molecular subtypes have different clinical courses, and there is insufficient data regarding predictive factors of response to available treatments for BC, beyond targeted therapies for hormonal and human epidermal growth factor 2 (HER2) receptors, programmed death-ligand 1 (PD-L1), and some somatic or germline driver mutations (Cardoso et al., 2019). Among these factors, the human microbiota could be a crucial piece of the puzzle to better anticipate individual BC risk and prognosis, pharmacokinetics, pharmacodynamics, and clinical efficacy (Roy and Trinchieri, 2017).

In this review, we discuss concepts related to the origin, composition, and evolution of human microbiota throughout life, the links between gut/breast microbiota and BC, and explore potential implications of metabolomics and pharmacomicrobiomics that might impact BC risk, prognosis, and possibly treatment choices toward a more personalised medicine. Finally, we put in perspective the potential limitations and biases regarding the current microbiota research and provide insights for new and more robust studies in this promising and challenging field.

## Discussion

### Gut Microbiota—Origin and Dynamic Evolution During the Human Life Cycle

Colonisation of the gut with microbes early in life is essential for the development of newborn’s immune system, metabolic function and potentially future health. There is accumulating evidence that intrauterine human foetal environment is not sterile and that placental microbial flora includes non-pathogenic commensal microbes of phyla Tenericutes, Firmicutes, Bacteroidetes, Proteobacteria, and Fusobacteria (Aagaard et al., 2014). A study in 50 women undergoing non-emergency caesarean sections reported that meconium and amniotic fluid microbiota were dominated by reads that mapped to *Pelomonas puraquae* and typical skin commensals, including *Propionibacterium acnes* and *Staphylococcus* spp., respectively. It was also demonstrated that bacterial DNA and short-chain fatty acids (SCFA) were present *in utero*, with a potential contribution to foetal immune system development (Stinson et al., 2019). This “*in utero colonisation*” contribute to a possible heritage of mother’s microbiota.

Immediately after birth, newborns experience rapid colonisation by other microorganisms, from their mothers and surrounding environment. Vaginally delivered babies acquire bacteria resembling maternal vaginal microbiota (predominantly *Lactobacillus* and *Prevotella*), whereas those born by caesarean acquire bacteria resembling the skin microbiota (mainly *Staphylococcus* spp.) (Stewart et al., 2018).

The evolution of gut microbiota develops in three different phases that occur throughout childhood: during the first year of life, the developmental phase, is when most microbial composition and diversity is defined if breastfed, as breast milk has higher levels of *Bacteroides* spp. and is associated with increased gut diversity and faster maturation; a transitional phase during the second year of life, with a contribution of different factors such as household exposures (e.g., siblings and furry pets), antibiotics and chronic conditions (e.g., asthma, allergies); and a stable phase on the third year of life and beyond that persists in adulthood and can be slightly modified by diet, lifestyle and diseases. In addition, geographic location is crucial for microbiota development, namely if the child is born in an urban or rural environment (Stewart et al., 2018).

After 65 years of age, microbiota enters a less diversified and stable state, resembling modifications observed in dysbiotic patients. This might contribute to the appearance of some diseases associated with ageing. In fact, it may reflect the cumulative impact related to lifestyle on the diversity of flora (Maynard and Weinkove, 2018).

### Gut Microbiota Composition, Diversity, Resilience, and Dysbiosis Concepts

Commensal microbiota is composed of microorganisms (archaea, protozoa, fungi, viruses, bacteria) that colonise gastrointestinal tract and other areas of the body. Human intestinal microbiota is estimated to contain 10^13^ bacterial cells, 10 times the number of human cells in the body. Bacterial load, along with species diversity, increase from the stomach to the colon, creating a complex microbial ecosystem (Roy and Trinchieri, 2017; Sommer et al., 2017; Stewart et al., 2018).

For microbiota analysis, it is important to know the principal concepts that allow us to understand the differences between a homeostatic and a dysbiotic microbiota (α and β-diversity). *Alpha*-diversity can be described as the richness of microbes present in the gut and can be calculated by Operational Taxonomic Units (OTU) count (which means the number of different species in the gut) and Shannon index (which measures how evenly the microbes are distributed in the gut). β-diversity is used to compare samples and assesses how different the microbial community is from one environment to another, like a microbiological fingerprint (Sommer et al., 2017).

Microbiota holds the capacity of self-regeneration, known as resilience, explained by the ability to restore its homeostasis after an external disorder (e.g., infections or antibiotic treatment). These modifications can be a pulse perturbation (present in a short period of time as antibiotic intake), or a press disturbance (continuous maintained process for an extended period of time, at a constant level, such as permanent diet changes or moving to another location) (Sommer et al., 2017).

Moreover, it is crucial to understand that resilience of microbiota depends on three variables for dynamic stability: time (remains the same microbiota composition through time and in response to perturbances), taxonomic groups (persistence of groups through perturbations or over time) and functional groups (although species and taxonomic groups may change, the functional properties of the microbiota remain the same). Only when an accumulated stress threshold is exceeded, a shift from homeostasis to a new balance occur (Sommer et al., 2017).

Resilience comprises four components: resistance, latitude, precariousness, and panarchy. Resistance is the capacity to remain the same during an external perturbation. Latitude can be defined by the maximum extent that a system can be pushed by a perturbation before the self-renewal capacity is lost. Precariousness comprehends the distance between initial state of equilibrium and a threshold of non-return. Finally, panarchy refers to how the intrinsic organisational state of microbiota can cope with stress (Sommer et al., 2017).

However, in some cases, after a perturbation, microbiota cannot remain resilient, which leads to a new equilibrium state, often named “dysbiosis.” Dysbiosis is a term that describes a diseased state of intestinal microorganism communities, which conducts to an intestinal-microbial imbalance in the host. Commonly, it is associated with a reduction of α-diversity and a low-grade of spontaneous inflammation of the mucosal barrier. That is why this condition correlates with a wide range of human diseases and can occasionally develop a state of high resilience to external perturbations (Sommer et al., 2017).

*Alpha* and β-diversity of microbiota are drivers of resilience potential. There are four mechanisms related to resilience capacity: dispersal, diversification, drift and selection. As an example, the mode of delivery (vaginal or caesarean) can cause dispersal (due to the impact on maternal-foetal transmission of microbiota), but also selection (through enhancement or decrement of the pool of microorganisms that foetus can select from). Therefore, dispersal means a new genetic variation, either by mutations or by introduction of a new organism. Diversification stands for migration of organisms through space. Drift corresponds to stochastic changes that can occur over time. Finally, selection has the same meaning as in nature, the survival of the fittest (Sommer et al., 2017).

### Interplay Between Gut and Breast Microbiota With Breast Cancer

Gut dysbiosis appears to be associated with increased risk of developing inflammatory, autoimmune, and malignant diseases, including BC. Dysbiosis can potentially favour oncogenesis and tumour progression and affect responses and toxicity profile of antineoplastic agents (Dzutsev et al., 2015; Lobionda et al., 2019; Nogueira and Shoenfeld, 2019).

Although current knowledge of links between microbiota and BC are still scarce, it is already possible to perceive several steps of this intriguing interaction. First, we must understand gut microbiota as if it were a metabolite-secreting gland with paracrine and systemic effects. For local metabolites, the classic example is related to microbiota influence on the transition from dysplasia to carcinoma. For systemic metabolites, their effect on distant cells is comparable to the action of hormones on their effector organs. Second, these hormone-like metabolites play an integral role in modulating various aspects of host physiology involving factors that comprise the “Hallmarks of Cancer.” Third, the cancer microenvironment *per se* can enhance the procarcinogenic activity of microbiota (Hanahan and Weinberg, 2011; Fulbright et al., 2017).

#### Breast Cancer Microbiota and Normal Breast Tissue Microbiota

Microbiota of human breast tissue is diverse and distinct from other areas of the organism regardless of the sample site within the breast, age, geographical area and pregnancy history. Diversity of local breast flora is not comparable to other microbial compartments (e.g., gut, skin, vagina). The unique breast microbiota pattern consists, in decreasing order, of Proteobacteria, Firmicutes, Actinobacteria, and Bacteroidetes. Although breast tissue microbiota has only recently began been evaluated, the presence of specific microbiota in human milk has already been known for several years. Also, composition of human milk microbiota seems to be similar to breast microbiota: Proteobacteria is the most abundant phylum, followed by the others phyla (Costantini et al., 2018; Zhang et al., 2020).

Bacterial microbiota of breast tissue and breast milk influences development of healthy infant gut microbiota and women’s health. Thus, breast dysbiosis may lead to lactational mastitis and can further influence metabolic development during breastfeeding period, adequate microbial colonisation, immune system maturation and infant growth. An example of this is the presence of butyrate-producing bacteria in human milk, such as *Roseburia* spp., *Coprococcus* spp., and *Faecalibacterium prausnitzii*. These genera can modulate the risk of childhood obesity, allergic diseases, and cancer (Prentice et al., 2019; Zhang et al., 2020).

Despite some controversy, current evidence supports that microbiota of human BC tissue is different from normal paired tissue. It is not known whether these findings reflect local dysbiosis, creating an environment that favours breast tumour formation (oncogenic trigger), or the natural selection of microorganisms adapted to a microenvironment rich in fatty acids and other local metabolites.

A study found that *Methylobacterium radiotolerans* was relatively abundant in oestrogen positive tumour tissue, and *Sphingomonas yanoikuyae* was relatively more prevalent in paired normal tissue. Moreover, total bacterial DNA load was lower in tumour tissue and advanced-stage BC when compared with healthy tissue and early stage BC, respectively. Additionally, lower baseline levels of antibacterial response gene expression in tumoural vs. healthy breast tissue was observed (Xuan et al., 2014). Another study pointed out for significant differences between sentinel cancer lymph nodes and normal samples for the presence of *M. radiotolerans* (Yazdi et al., 2016). However, abundance of this bacteria in BC was countered in another investigation (Wang et al., 2017). In an Asiatic cohort of patients, an increased representation of genus *Propionicimonas* and the families *Micrococcaceae*, *Caulobacteraceae*, *Rhodobacteraceae*, *Nocardioidaceae*, and *Methylobacteriaceae* was observed in BC tissues. Nevertheless, caution should be taken on extrapolation for a Western population (Meng et al., 2018). In a Canadian study, it was reported that adjacent normal tissue of women with BC had a higher relative abundance of *Bacillus*, *Enterobacteriaceae*, and *Staphylococcus*, compared to normal breast tissue of healthy controls (Urbaniak et al., 2016). The different β-diversity between BC and healthy controls was also confirmed in nipple aspirate fluid (NAF), which may have a potential role in carcinogenesis. It was noticed that there were differences in the composition of microbiota and in functional properties associated with identified bacteria (Chan et al., 2016).

Conversely to previous data, a study with a Mediterranean population found more similarities than differences between tumours and adjacent normal tissues. Yet, supporting the variability, it was determined for the first time the presence of genus *Ralstonia*, phylum Proteobacteria, as the most abundant genus found in breast tissue. Following *Ralstoni*a in terms of abundance, the presence of genera *Methylobacterium* and *Sphingomonas* in phylum Proteobacteria was confirmed consistent with previous studies (Costantini et al., 2018).

These findings may reflect the influence of bacteria and/or their components in local immune microenvironment, highlighting unrecognised links between breast dysbiosis and BC that might exist.

#### Gut Microbiota as a Metabolite-Secreting Gland With Effects on Breast Tissue

Gut microbiota metabolises and secretes hormone-like bioactive compounds that modulate BC risk, such as reactivated oestrogens, active phytoestrogens, SCFA, lithocholic acid (LCA) and cadaverine.

Plottel and Blaser defined human estrobolome as “the aggregate of enteric bacterial genes whose products are capable of metabolising oestrogens” (Plottel and Blaser, 2011). An important role of intestinal microbiota is the modulation of systemic oestrogens, as microbes and its’ metabolites affect enterohepatic circulation of oestrogens and their resorption. Theoretically, an estrobolome enriched with enzymes such as β-glucuronidase (BGUS), which is produced by many intestinal bacteria, could play a major role in the deconjugation of xenobiotics and sexual hormone oestrogens. This leads to their reuptake via enterohepatic pathway and, therefore, increases the time they remain in the body. In particular, resorption of free oestrogen produced by BGUS may increase the risk of hormone-dependent BC in women. The bacterial genera *Escherichia* and *Shigella*, members of the phylum Proteobacteria, have BGUS activity. It is likely that bacterial composition of estrobolome is affected by host factors (e.g., age and ethnicity), as well as environmental influences throughout life, including diet, alcohol and use of antibiotics, which can exert selective pressures on bacterial populations. Some of these factors were also independently associated with increased risk of BC (Plottel and Blaser, 2011; Kwa et al., 2016). A population-based case-control pilot study in postmenopausal women showed that those with BC had a statistically significant modified composition (β-diversity) and lower oestrogen-independent α-diversity, which means less microbial richness and diversity (Goedert et al., 2015).

Hence, oestrogen metabolism–gut microbiota axis dysfunction combined with underlying individual variations in oestrogen levels may contribute to an increased risk of hormone-driven malignancies, including BC. In the future, interventions involving the use of prebiotics, probiotics, symbiotics, and/or antimicrobial agents may be considered. These therapeutic strategies can be specifically designed to modulate intestinal bacterial populations with BGUS activity to decrease the risk of oestrogen-related BC, or after cancer diagnosis, become complementary treatments (Plottel and Blaser, 2011; Kwa et al., 2016).

From another perspective, specific types of intestinal bacteria are able to metabolise phytoestrogens (e.g., isoflavones and lignans) and convert them into active metabolites that can protect against BC. Interventional studies have shown that the ability of some individuals’ intestinal microbiota to convert isoflavones and lignans into equol and enterolignans, respectively, may result in a reduced risk of hormone-dependent diseases, such as BC. In addition, some isoflavones are absorbed only in the colon after being metabolised by the intestinal microbiota. Soy isoflavones are composed of daidzine, genistin and glycitine, and their absorption is highly dependent on β-glucosidase-producing bacteria (e.g., lactic acid bacteria). Due to the activity of this enzyme, daidzine is transformed into daidzein, which is converted into dihydrodaidzein. The latter metabolite is usually transformed into *O*-desmethylangolensin (*O*-DMA), but in a smaller percentage of the population it is converted to 7-hydroxy- 3-[49-hydroxyphenyl]-chroman (equol). However, not all individuals consuming isoflavones produce equol. Generally, 20–30% of Western population convert this isoflavone to equol (as opposed to 50–60% of Asian population) (Yuan et al., 2007; Poluzzi et al., 2014; Gaya et al., 2016).

In terms of beneficial effects and when comparing all isoflavones and their metabolites, equol is the most potent estrogenic metabolite, owing to its higher affinity for oestrogen receptors, unique antiandrogenic properties and superior antioxidant activity. The antioxidant potential of equol may be a result of its non-planar structure, which provides higher flexibility for conformational changes and allows better penetration into the membrane. Furthermore, it can prevent oxidative damage *in situ*, as opposed to other isoflavones, that hold a more rigid structure (Yuan et al., 2007). Most of equol-producing bacteria isolated belong to *Coriobacteriaceae* family, such as genera *Adlercreutzia*, *Assacharobacter*, *Eggerthella*, *Enterorhabdus*, *Paraeggerthela*, and *Slackia.* Among these, species such as *Adlercreutzia equolifaciens, Asaccharobacter celatus, Enterorhabdus mucosicola, Slackia isoflavoniconvertens*, and *S. equolifaciens* are reported to be equol producers (Yuan et al., 2007).

In the gut, bacterial fibre fermentation results in generation of free fatty acids (FFAs) of various chain lengths, a class of nutrients which are sensed by specific membrane-bound FFA receptors (FFARs), expressed exclusively throughout gut. In particular, FFAR1 (GPR40) and FFAR4 (GPR120) are activated by medium and long-chain saturated and unsaturated FFAs, while FFAR2 (GPR43) and FFAR3 (GPR41) are activated by SCFA, mainly acetate, butyrate, and propionate. FFARs have an important role on physiological functions such as production of peptide YY, glucose stimulated-insulin secretion, regulation of inflammatory mediators, leptin production, among others (Thirunavukkarasan et al., 2017).

Emerging data suggest a role of these receptors in normal and pathological conditions, including BC. It has been proposed that FFAR2 and FFAR3 might participate in tumour suppression through propionate and butyrate, influencing cell proliferation and inducing apoptosis (Thirunavukkarasan et al., 2017). In a study with BC cells lines, evaluating the interplay between FFARs and propionate and butyrate, it was observed that FFAR2 could increase the levels of E-cadherin in mesenchymal-like cells but not on epithelial-like cells, which suggests this receptor might convert cells to an epithelial phenotype, impacting epithelial-mesenchymal transition (EMT) and potentially decreasing risk of metastases (Thirunavukkarasan et al., 2017). On the other hand, FFAR3 has been shown to avoid invasion, but with a different mechanism. FFAR3 relies on decreasing ERK phosphorylation, affecting MAPK/ERK1/2 pathway, which is responsible for regulation of transcription factors fundamental for the EMT process (Thirunavukkarasan et al., 2017). Thus, microbiota can have a relevant influence in determining invasive phenotypes of BC, due to the fact that a percentage of bacteria with anti-inflammatory characteristics, such as *Akkermansia muciniphila* and *Faecalibacterium prausnitzii*, can produce SCFAs in a relatively abundant quantity (Lukovac et al., 2014). However, these conclusions should be taken carefully, because this organoid model experimental study does not consider variables such as host and tumour microenvironment, namely on the bioavailability of SCFAs and the expression of FFAR2 and FFAR3.

Generally, bile acids (BAs) are known to be involved in lipid metabolism, cholesterol elimination, bile flow and cholesterol biosynthesis. More recently, it was noticed that BAs also directly or indirectly interfere as a signalling molecule in cell proliferation, metabolism and differentiation (Hanafi et al., 2018; Mikó et al., 2018).

BAs are derived from cholesterol and are stored in the gallbladder. Cholic acid (CA) and chenodeoxycholic acid (CDCA) are the two most common types of primary BAs. They are synthesised and conjugated in the liver into bile salts, such as glycocholic acid (GCA), taurocholic acid (TCA), glycochenodeoxycholic acid (GDCA), taurochenodeoxycholic acid (TDCA) and ursodeoxycholic acid (UDCA). Cytotoxicity of BAs is dependent on their structural formation, while their hydrophobicity is based on number and position of the hydroxyl group in the ring structure. UDCA, the most hydrophilic BA, seems to play a role, mediated by sphingomyelinase, in proapoptotic signalling in cancer cells, such as gastric, prostate and colon cancer cells. The most common secondary BAs, deoxycholic acid (DCA) and LCA (the most hydrophobic BA), are synthesised by bacterial microbiota of the small intestine. These two BAs are produced as a result of deconjugation and dihydroxylation of primary BAs. Increased serum levels of hydrophobic BAs, such as CDCA and DCA, have been associated with colon cancer, gallstones, and other gastrointestinal diseases. However, DCA or UDCA have no effect on BC cells at normal range concentrations (Hanafi et al., 2018; Mikó et al., 2018).

On the other hand, LCA has antiproliferative action in BC. Bacterial enzyme catalysing the formation of LCA is 7α/β-hydroxysteroid dehydroxylase (baiH). Anaerobic bacteria, mainly the Clostridiales, are responsible for this BA transformation. LCA, through Takeda G-protein coupled receptor (TGR5), exerts antineoplastic effects on BC cells by inhibiting EMT, endothelial growth factor vascular production, metastasis formation, induced antitumour immunity and changes in metabolism. Moreover, bacterial machinery for LCA biosynthesis is decreased in the early stage BC, and serum levels of LCA correlate negatively with Ki67 marking index in BC (Mikó et al., 2018). Although human cells are also capable of synthesising cadaverine, production of bacterial cadaverine is dominant over human biosynthesis. Cadaverine is synthesised from lysine by bacterial enzymes LdcC and CadA. *Shigella flexneri*, *S. sonnei*, *Escherichia coli*, and *Streptococci* have been shown to express cadaverine biosynthetic enzymes. Cadaverine seems to have effects in cellular proliferation, EMT, cellular migration, invasion, and tumour infiltration to surrounding tissues. In addition, cadaverine alters metabolism in BC cells and reduces proportion of aldehyde dehydrogenase 1 (ALDH1+) cancer stem cells in 4T1 murine BC cells (Kovács et al., 2019; Mikó et al., 2019). In a cohort of triple negative BC (TNBC) patients, cell marker ALDH1 was correlated with aggressive biology of BC (high histological and nuclear grades) (Nalwoga et al., 2010). The trace amine-associated receptor-1 (TAAR1), one of the receptors from which the cadaverine performs its functions, was already associated with inhibition of BC growth. In parallel to what was described for LCA, cadaverine is decreased in BC, most dominantly in early stage (Kovács et al., 2019; Mikó et al., 2019).

#### Dynamic Evolution of Gut and Breast Microbiota Through Breast-Gut Axis

There are many questions that remain unanswered which are relevant to fully comprehend the relationship between human microbiota with BC: what is the true origin of breast microbiota? Is there an established gut-breast axis? What are the links between gut and breast microbiota and BC oncogenesis–the so-called “oncobiome”? Some authors advocate a potential role of bacterial uptake in the intestinal mucosa mediated by dendritic cells through an endogenous route as the potential source of breast microbiota; others suggest the existence of bloodstream transmission of translocated bacteria from intestinal epithelium that quickly reach the breast (Rescigno et al., 2001; Costantini et al., 2018; Zhang et al., 2020).

Gut and breast dysbiosis are probably a reflection of exogenous factors (e.g., hormone replacement therapy, heavy alcohol use), but also a result of the influence of endogenous factors related to the host itself (e.g., ethnicity, overweight and obesity, menopausal state, BRCA1/BRCA2 genetic mutations), many of them established risk factors for BC (Cardoso et al., 2019).

Gut microbiota of premenopausal and postmenopausal women is different and produces distinctive metabolites. Intestinal microbiota species that act synergistically in premenopausal period tend to compete with each other in postmenopausal period. As an example, in postmenopausal women, a decrease in calcium absorption can trigger a fierce competition between different species for the same substrate (Zhao et al., 2019). The data supports a lower Firmicutes/Bacteroidetes (F/B) ratio and lower relative abundance of *Lachnospira*, *Roseburia* spp. in postmenopausal women. In contrast, a higher relative abundance of genera *Prevotella*, *Tolumonas*, *Parabacteroides*, and *Bilophila* is present during the postmenopausal period (Santos-Marcos et al., 2018; Zhao et al., 2019). A comprehensive shotgun metagenomic analysis confirmed previous data suggesting a difference between postmenopausal BC patients and healthy controls in composition and functions of gut microbial community (Goedert et al., 2015; Zhu et al., 2018). In this study, several bacterial species were found to be enriched in postmenopausal BC patients: *E. coli*, *Citrobacter koseri*, *Acinetobacter radioresistens*, *Enterococcus gallinarum*, *Shewanella putrefaciens*, *Erwinia amylovora*, *Actinomyces* spp. *HPA0247*, *Salmonella enterica*, and *Fusobacterium nucleatum*. In contrast, other species were less abundant in this subgroup: *Porphyromonas uenonis*, *Eubacterium eligens*, *Roseburia inulinivorans*, and *Lactobacillus vaginalis.* Interestingly, in postmenopausal BC patients there was a decrease in butyrate-synthesis genes expression, potentially related to low abundance of butyrate-producing bacteria (e.g., *R. inulinivorans*). As mentioned earlier, this may have contributed to chronic inflammation and increased risk for BC (Zhu et al., 2018).

Obesity affects more than half of postmenopausal women and is associated with increased total fat mass and abdominal fat and decreased lean body mass compared with premenopausal women, regardless of ageing (Vieira et al., 2017). Percentage of body fat seems to be intrinsically inversely related to *A. muciniphila* abundance in BC patients. Noteworthy, BC women with high relative abundance of *A. muciniphila* have higher levels of *Prevotella*, *Lactobacillus*, and lower levels of *Clostridium*, *Campylobacter* and *Helicobacter* compared to patients with low abundance of the former (Frugé et al., 2020). Moreover, the significance of F/B ratio in overweight/obesity is still controversial across the studies, with numerical variations in Firmicutes abundance. A recent study reported a decline in total bacteria load in overweight and obese patients when compared with normal body mass index (BMI) patients. A significantly lower number of total Firmicutes, *F. prausnitzii*, *Blautia* spp., and *Eggerthella lenta* was observed in patients with overweight/obesity. In the same study, a correlation between gut microbiota and BC clinical stages/histoprognostic grades was performed. Percentage and absolute numbers of *Clostridium coccoides* cluster, *C. leptum* cluster, *F. prausnitzii* were significantly higher in clinical stage II/III than in clinical stage 0/I, while *Blautia* was associated with a higher histoprognostic grade (Luu et al., 2017).

Even ethnicity seems to have an important role in BC risk. African descendants’ patients have a higher probability of developing more biologically aggressive forms of BC with lower survival rates such as TNBC molecular subtype (Saraiva et al., 2017). There are several factors contributing to BC racial disparities. African women tend to have higher BMI than Caucasians, and there is a link between obesity and BC, including TNBC (Carey et al., 2006; Saraiva et al., 2017). Moreover, there is data suggesting that TNBC in black women has more active signalling via insulin growth factor receptor type 1 (IGF1), Wnt/ß-catenin pathways and more ALDH1 expression (Nalwoga et al., 2010; Saraiva et al., 2017). In addition, there are potential microbiotic factors that may interact with BC biology. A recent study identified racial differences in breast microbiota between non-Hispanic black (NHB) and non-Hispanic white (NHW) women and also detected distinct microbiota patterns in relation to stage and BC subtype: genus *Ralstonia* was most abundant in BC of NHB woman, whereas family *Xanthomonadaceae* was more abundant in BC of NHW women (both belong to phylum Proteobacteria); phylum Bacteroidetes was significantly lower among NHB as compared to NHW; enrichment of family *Streptococcaceae* in TNBC; higher abundance of genus *Bosea* (phylum Proteobacteria) increased as stage advanced (Smith et al., 2019). However, besides predisposing factors associated with African ancestry, it is important to reinforce that black-white disparities in BC may also be explained by demographics and socioeconomic factors (Curtis et al., 2008).

Gut and breast microbiota might also modulate tumour microenvironment. The “Hallmarks of Cancer,” proposed by Hanahan and Weinberg in 2001 and updated in 2011, logically defined how a normal cell progresses to a tumourigenic state within a complex neoplastic environment (Hanahan and Weinberg, 2011). Thus, breast oncogenesis turns out to be a sequential or concomitant influence of several biological steps contributing to evolution of normal tissue to dysplasia and invasive cancer. In this complex interplay, microbiota, being one of the protagonists of that “local habitat,” seems to have a (relevant) role in several mechanisms of BC carcinogenesis: activating signals for aberrant epithelial proliferation; secretion of growth factors by senescent cells; genome instability and mutations; perturbations to local metabolic microenvironment; specific microbes influence angiogenesis and tumour-associated remodelling of the vasculature; avoiding immune destruction; tumour-promoting inflammation (Hanahan and Weinberg, 2011; Fulbright et al., 2017).

Bacteria and their metabolites can modulate different signalling pathways, including the Wnt/ß-catenin. This pathway has a crucial role in maintenance of adult tissue homeostasis by regulating cell proliferation, migration, differentiation, survival and adhesion, and renewal of stem cells (Fulbright et al., 2017; Sferrazza et al., 2020). *Bacteroides fragilis* enterotoxin promotes cleavage of E-cadherin, an intercellular adhesion molecule that plays a major role in maintaining intestinal epithelial homeostasis in basal and inflammatory conditions (Rhee et al., 2009; Fulbright et al., 2017). *Fusobacterium nucleatum* also leads to deregulation of this pathway, through adhesin FadA that binds E-cadherin and promotes cell proliferation (Rubinstein et al., 2013; Fulbright et al., 2017). As a result of the inactivation of E-cadherin, there is a nuclear translocation of ß-catenin, transcription of proto-oncogene c-Myc, and cellular hyperplasia (Fulbright et al., 2017). Another pathway that may be involved in chronic inflammation of breast tissue and in BC progression is related to human papillomavirus (HPV) infection and viral induction of transcription 3 (STAT3) signalling and consequent elevation of interleukin 17 (IL-17). These data show that gut and breast microbiota can activate signals for aberrant cell proliferation (Zhang et al., 2016). Persistent dysregulation of the DNA damage response and repair in cells causes genomic instability. The resulting genetic changes allow pre-malignant and malignant cells to retain and accelerate the rate of mutations (Fulbright et al., 2017; Palovcak et al., 2017). There is evidence that *E. coli*, *Staphylococcus epidermidis*, and *E. faecalis* have the ability to inflict DNA damage through production of genotoxins (including colibactin), hydroxyl and superoxide radicals (Urbaniak et al., 2016; Eslami-S et al., 2020). In addition, *E. coli* colibactin can induce senescence-associated secretory phenotype of growth factors capable of stimulating cell proliferation and tumour progression (Fulbright et al., 2017; Chadha et al., 2020).

Perturbations to gut and breast metabolic environment can also favour or inhibit BC oncogenesis.

As discussed earlier, one of the ways gut and breast microbiota could influence tumourigenesis is by enhancing local exposure of breast tissue to a hormonal trigger. Endogenous progesterone metabolites may provide a new hormonal basis for BC. BC tissue has elevated 5α-reductase activity, which results in significantly higher total levels of 5α-pregnanes, especially 5α-pregnane-3,20-dione (5αP), whereas normal breast tissue produces more 4-pregnenes, especially 3α-hydroxy-4-pregnen-20-one (3αHP). Thus, local changes in progesterone metabolism, with an increased 5α-pregnane:4-pregnene (especially 5αP:3αHP) ratio, may promote BC by stimulating increased cell proliferation and detachment. Increases in 4-pregnenes may retard these tumourigenic processes (Wiebe et al., 2000). One of possible local mechanisms in tumour microenvironment that might lead to increased production of 5αP is higher levels of *Bacillus cereus*. This Gram-positive agent was found to be elevated in BC tissue. This has already been shown to promote proliferation of tumour cells *in vitro* by metabolising progesterone to 5αP (Urbaniak et al., 2016; Chadha et al., 2020).

Polycyclic aromatic hydrocarbons such as oestrogens and other hormones are linked to BC development. Metabolic pathway related to BGUS activity appears to be associated with increased risk of hormone-dependent BC (Plottel and Blaser, 2011; Kwa et al., 2016). Oestrogen is detoxified by conjugation with glucuronidate to become soluble. When this conjugated oestrogen reaches a site with increased BGUS activity, this enzyme leads to accumulation of biologically active oestrogens which have a carcinogenic role. Intriguingly, positive BGUS bacteria are present and increased in local BC microbiota (even in NAF of BC survivors) (Thompson et al., 2017). *Streptococcus pyogenes* is capable of increasing oestrogen levels in mammary tissue through BGUS activity (Thompson et al., 2017). Conversely, *S. yanoikuyae* has the ability to metabolise oestrogens locally, potentially constituting a protective factor in healthy controls opposite to what is observed in patients with BC (Xuan et al., 2014). In addition to E-cadherin, SCFAs are considered essential for regulation of intestinal epithelial integrity and host cellular metabolism. These products are among the most important of bacterial metabolites and have long been recognised as relevant substrates for preserving a healthy gut (Chambers et al., 2018). SCFAs modulate numerous cancer hallmarks, such as cell proliferation, apoptosis, cell invasion, gene expression, and metabolism in BC (Mikó et al., 2019). Otto Warburg observed that cancer express high rates of fermentation in the presence of oxygen (Warburg et al., 1927). This “Warburg Effect” is linked to mitochondrial dysfunction and genetic mutations within the cancer cell (Warburg et al., 1927; Mikó et al., 2019). In this way, BC cells rely heavily on glucose for energy (Warburg et al., 1927; Fulbright et al., 2017). In BC cells, SCFAs can be used directly as energy substrates and in particular butyrate can lead to oxygen consumption, inhibition of lactate metabolism and slowed cell proliferation (Fulbright et al., 2017; Mikó et al., 2019). Furthermore, increases in intracellular butyrate will act as a histone deacetylase inhibitor, promoting apoptosis and inhibiting cell proliferation through epigenetic modifications (Fulbright et al., 2017). As discussed earlier, bacterial machinery for LCA and cadaverine biosynthesis is decreased in the early stage BC (Kovács et al., 2019; Mikó et al., 2019).

Moreover, besides metabolic activity, bacteria can interfere with immune system’s response. Ductal epithelium can detect microbial-derived signalling through Toll-like receptors (TLR) and nucleotide-binding oligomerisation domain (NOD)-like receptors (NLRs). While some TLRs may be procarcinogenic, such as TLR2-MyD88, others may play a protective role, inhibiting growth of cancer cells and having potent antitumoural activity, such as TLR5 (Scheeren et al., 2014; Chan et al., 2016). As an example, it was demonstrated that *S. yanoikuyae* could activate TLR5 using a single polar flagellum as a ligand for TLR5 (Chan et al., 2016). As mentioned earlier, HPV stimulates IL17-driven inflammation and contributes to an inflammatory and procarcinogenic microenvironment. Likewise, *Alistipes* through IL-6 and STAT3 activation, induces epithelial hyperplasia and barrier dysfunction (Zhang et al., 2016; Fulbright et al., 2017). Recently, it was documented that *F. nucleatum* reaches breast tissue via haematogenous route and specifically attaches to breast cells trough a bacterial lectin-host sugar (Fap2-Gal-GalNAc) interaction. Furthermore, colonisation of breast tissue by *F. nucleatum* is secondary to tumour initiation, using Fap2 adhesion to silence cytotoxic immune cells and reduce infiltration of CD4 + and CD8 + T cells into “infected tumours.” The data also support that *F. nucleatum* accelerates tumour growth and metastatic progression (Parhi et al., 2020).

In another investigation, it was also demonstrated that *Listeria fleischmannii* and *Neisseria subflava* were correlated with cancer gene expression, mainly genes involved in EMT. Within the same analysis, *Haemophilus influenzae* was correlated with genes representing crucial pathways for tumourigenesis: G2M checkpoint, E2 signalling, and mitotic spindle assembly. Interestingly, an increase in the presence of *H. influenza*e was found in non-cancerous adjacent samples, suggesting that oxidative tumour environment is harmful, forcing bacteria to reside predominantly in surrounding stromal tissue. These results are in line with previous data demonstrating that non-cancerous adjacent tissue represents an intermediate state between healthy and invasive disease (Thompson et al., 2017).

The vascular network is important since tumour persistence and proliferation depends on an adequate supply of oxygen, nutrients, and removal of catabolic products. However, links between microbiota and angiogenesis are limited. Microbiota is necessary for normal development of vasculature in gut and, during an infectious process, there is an interaction between microbiota metabolites and TLR to promote angiogenesis. However, the same behaviour for tumour-induced angiogenesis cannot be predicted (Fulbright et al., 2017).

Finally, regarding molecular subtypes, unique and common BC complete microbiota signatures (bacteria, viruses, fungi, and parasites) have been identified (Costantini et al., 2018; Smith et al., 2019). Oestrogen receptor-positive and HER2 positive diseases share a more similar microbial signature than TNBC tissues. Main signatures common to all four molecular types identified were Proteobacteria, Firmicutes, Actinobacteria, and Bacteroidetes (Costantini et al., 2018).

#### Gut Microbiota and Its Impact on Breast Cancer Treatment

Microbiota affects chemo-, hormone-, targeted-, immuno-, and radio-therapy for BC. In recent years, it has been shown that gut microbiota may modulate cancer treatments’ efficacy and adverse effects. On the other hand, it is also apparent that both cancer itself and anticancer therapies interact with gut microbiota bidirectionally (Supplementary Material; Panebianco et al., 2018; Wilkinson et al., 2018; Gately, 2019).

Pharmacomicrobiomics is defined as the effect of microbiome variations on drug disposition, action and toxicity (ElRakaiby et al., 2014; Panebianco et al., 2018). Here, we present the most relevant knowledge to date regarding the impact of microbiota in drugs frequently used to treat BC patients.

A study reported that under anaerobic conditions, a group of bacteria from *Enterobacteriaceae* family have the ability to inactivate doxorubicin, influencing its bioavailability and toxicity profile. *Raoultella planticola* deglycosylates doxorubicin into 7-deoxydoxorubicinol and 7-deoxydoxorubicinolone through reductive deglycosylation. Degradation of doxorubicin by *Klebsiella pneumoniae* and *E. coli* BW25113 is dependent on molybdopterin-dependent enzymes (Yan et al., 2018).

Cyclophosphamide, in addition to antimitotic and antireplicative effects, has immunosuppressive and immunomodulatory properties. In tumour-bearing mice, it leads to disruption of intestinal barrier and migration of a set of Gram-positive species (*L. johnsonii*, *L. murinus*, and *E. hirae*) to mesenteric and spleen lymph nodes. Subsequently, there is a generation of inflammatory T helper 17 (Th17) and Th1 lymphocytes. Tumour-bearing mice that were germ-free or that had been treated with antibiotics against Gram-positive bacteria revealed a reduction in Th17 responses and resistance to cyclophosphamide treatment. Adoptive transfer of Th17 cells partially restored efficacy of this alkylating drug (Viaud et al., 2013; Panebianco et al., 2018). In a subsequent study, oral gavage with *E. hirae* was shown to restore the response to cyclophosphamide in tumour-bearing antibiotic-treated mice (Daillère et al., 2016; Panebianco et al., 2018).

Taxanes or taxoids are a closely related group of antineoplastic agents that stabilise cellular microtubules. Recent investigation supports the hypothesis that paclitaxel could decrease the count and capacity of beneficial gut bacteria, interfering with proper functioning of intestinal barrier and increasing systemic exposure to bacterial products and metabolites. In this setting, for example, a decrease in the levels of *A. muciniphila* can be a driving factor for systemic inflammation and taxanes-induced neuropathic pain. In the same study, it was also hypothesised that *L. intestinalis* and *E. siraeum* are inhibitors of the pain phenotype (Ramakrishna et al., 2019). In a randomised trial to detect the effect of symbiotics on adverse effects of combined chemotherapy (including docetaxel), a decrease in diarrhoea, lymphopenia and febrile neutropenia has been reported. In detail, the symbiotic preparation included *Bifidobacterium breve* strain Yakult, *L. casei* strain Shirota, and galactooligosaccharides (Motoori et al., 2017; Panebianco et al., 2018).

Gemcitabine (2′,2′-difluorodeoxycytidine) is a deoxycytidine analogue which inhibits DNA synthesis. It was found that bacteria, mainly belonging to the class Gammaproteobacteria, can metabolise gemcitabine in its inactive form (2′,2′-difluorodeoxyuridine). This metabolism is dependent on the expression of a long isoform of bacterial enzyme cytidine deaminase (CDDL), observed primarily in this class of bacteria. In a cancer mouse model, gemcitabine resistance caused by intratumoural Gammaproteobacteria was reversed by co-administration of ciprofloxacin, thus supporting the role of these bacteria in the failed response to this drug (Geller et al., 2017; Panebianco et al., 2018). *Mycoplasma* spp. (including *M. hyorhinis*) colonise tumour tissue in cancer patients. Presence of this bacteria could be a limiting factor for gemcitabine efficiency by expressing nucleoside analogue-catabolising enzymes (pyrimidine nucleoside phosphorylase and CDDL) (Panebianco et al., 2018).

The importance of microbiota to modulate platinum salts response was also verified. The mechanisms of action depend not only on the formation of platinum-DNA adducts which block DNA replication and stimulate production of reactive oxygen species (ROS) and oxidative stress, but also on its ability to stimulate an immune response (Panebianco et al., 2018). To investigate whether microbiota changes tumour microenvironment, mice received an antibiotic cocktail (vancomycin, imipenem, and neomycin) starting 3 weeks before tumour inoculation and continuing throughout experiment. Antibiotic treatment affected tumour cytotoxicity shortly after chemotherapy, preventing platinum-induced DNA damage and apoptosis after DNA adduct formation by decreasing ROS production. This effect was documented using both oxaliplatin and cisplatin, the latter unable to trigger immunogenic cell death. Hence, data suggest that microbiota modulate platinum salts genotoxicity independently of an immunogenic response (Iida et al., 2013; Panebianco et al., 2018). Consistently with this data, an increase in tumour size and decrease in survival rate was observed in a mouse model with lung cancer receiving cisplatin combined with antibiotics, compared to animals receiving cisplatin alone. In contrast, mice administered with cisplatin combined with *Lactobacillus* showed a better response to therapy (Gui et al., 2015; Panebianco et al., 2018).

5-Fluorouracil (5-FU), 5-fluoro-2′-deoxyuridine (FdUrd) and 5-trifluorothymidine (F3(d)Thd) are metabolised to their corresponding active forms, which in turn inhibit DNA synthesis through thymidylate synthase (TS). Several studies suggest a selective death of commensal microbiota after 5-FU administration, allowing expansion of potentially pathogenic species and a decrease in F/B ratio (Li et al., 2017; Panebianco et al., 2018). Following 5-FU, an increase in *Clostridium* spp., *Staphylococcus* spp. and *E. coli*, and a decrease in *Bacteroides* spp., *Bifidobacterium* spp., *Lactobacillus* spp., and *F. prausnitzii* are reported in faecal microbiota (Panebianco et al., 2018). Furthermore, a study reported that faecal transplant (FMT) from healthy mice was able to prevent total body weight loss and colon shortening in 5-FU treated mice (Li et al., 2017). Enzymatic functions of specific bacteria also have a pivotal role in modifying the toxic profile of 5-FU and capecitabine (Alexander et al., 2017). *Mycoplasma hyorhinis* encodes a thymidine phosphorylase that significantly restricts cytostatic activity of pyrimidine nucleoside analogue compounds such as FdUrd and F3(d)Thd *in vitro*. Interestingly, the opposite is true for capecitabine (N4-pentyloxycarbonyl-5′-deoxy-5-fluorocytidine) metabolite 5-fluoro-5′-deoxyuridine (5’dFUrd), which is more effective in the presence of *M. hyorhinis*, as this prodrug is activated by the same enzyme (Alexander et al., 2017).

HER2 is amplified or overexpressed in 20–30% of all BC patients. It has been demonstrated that HER2 positive BC exhibits sensitivity to HER2 inhibitors, such as trastuzumab, pertuzumab, and trastuzumab emtansine (Cardoso et al., 2019; Cardoso et al., 2020; Yi et al., 2020). In a recent work, patients treated with neoadjuvant trastuzumab, who achieved pathological complete response (pCR) were characterised by higher abundance of Clostridiales bacteria and lower representation of Bacteroidales as compared to those with residual disease (RD) at surgery. When performing FMT from pCR and RD patients into recipient mice, it was replicated the treatment response observed previously in patients, indicating a causal role for commensal bacteria. Moreover, antibiotics abrogated trastuzumab benefit in mice, with decreased levels of Clostridiales bacteria and compromised recruitment of immune cells in tumour microenvironment (Martina, 2020). There is data reporting that the combination of *Lactococcus lactis* or *L. paracasei* with trastuzumab improved its efficacy in mice under vancomycin regimens (Martina, 2020). Furthermore, there is a case report of successful treatment of refractory pertuzumab-induced gastrointestinal toxicity with rifaximin, a non-absorbable oral antibiotic approved for irritable bowel syndrome with diarrhoea. Rifaximin inhibits small intestinal bacterial overgrowth and modulates gut microbiota. Following this pharmacological intervention, patient was able to continue and complete the planned treatment without dose reduction or delay (Soyano et al., 2017).

Regarding hormone therapy, letrozole, an aromatase inhibitor, was associated with a time-dependent shift in gut microbiota and a substantial reduction in overall species and phylogenetic richness. This treatment was correlated with a relative decrease in all of Bacteroidales OTUs and an increase in a majority of Firmicutes OTUs including 5 OTUs identified as *Lachnospiraceae*, one in family *Erysipelotrichaceae*, genus *Allobaculum* and two in family *Ruminococcaceae*. Only four Firmicutes OTUs significantly decreased after letrozole treatment (two Clostridiales: *Ruminococcaceae* and *Dehalobacteriaceae*).Notably, the shift in microbiota of letrozole-treated mice after 1 week of treatment was followed by significantly increased adiposity in second week of the experiment (Kelley et al., 2016). As mentioned previously, specific types of bacterial species are able to metabolise phytoestrogens and convert them into active metabolites that can protect against BC. There is data supporting that combination of daidzein with tamoxifen increased protection against breast carcinogenesis, while the combination of genistein with tamoxifen has an opposing effect when compared with tamoxifen alone (Constantinou et al., 2005).

A plethora of immunotherapy options is now part of treatment armamentarium of several malignancies, including BC (Alpuim Costa et al., 2020b). New generation immunotherapy is represented by the blockade of immune checkpoints, such as cytotoxic T-lymphocyte-associated antigen 4 (CTLA-4) and PD-(L)1, which are negative regulators of T cell proliferation and functions (Panebianco et al., 2018). In cancer mouse models, it was observed that germ-free animals or those treated with broad-spectrum antibiotics had no response to ipilimumab. Particularly, *Bacteroides thetaiotaomicron* and *B. fragilis* were identified as responsible for CTLA-4 blockade efficacy through the induction of a Th1 immune response (Panebianco et al., 2018; Vétizou et al., 2015). When analysing colitis secondary to anti-CTLA-4 treatment, there were differences between subjects prone and resistant to colitis: a higher abundance of bacteria from the Bacteroidetes phylum, specifically from *Bacteroidaceae*, *Rikenellaceae*, and *Barnesiellaceae* families, was observed in patients who did not manifest adverse events (Panebianco et al., 2018). Furthermore, a paucity of bacterial genetic pathways involved in polyamine transport and vitamin B biosynthesis has been found to be associated with an increased risk of developing ipilimumab-induced colitis (Dubin et al., 2016; Panebianco et al., 2018). In addition, if vancomycin was administered to mice with colitis and treated with anti-CTLA-4, they had more severe manifestation of intestinal disease, while administration of a common probiotic with *Bifidobacterium* could alleviate symptoms, without a detrimental effect on antitumour immunity (Dubin et al., 2016; Panebianco et al., 2018).

Several recent studies demonstrated an impact of gut microbiota in mediating response to anti-PD-(L)1 immunotherapy: oral administration of a cocktail of *Bifidobacterium* spp. combined with an anti-PD-L1 antibody almost abolished cancer growth (Sivan et al., 2015); commensal microbiota with abundance of *B. longum*, *Collinsella aerofaciens*, and *E. faecium* was associated with anti-PD-1 cancer efficacy (Matson et al., 2018); higher microbial diversity and increased levels of *Ruminococcaceae* in treatment responders and FMT from responders patients to germ-free animals enhanced response (Gopalakrishnan et al., 2018); faecal levels of *A. muciniphila* directly correlated with anti-PD-1 therapy efficacy and FMT from responders patients to germ-free or antibiotic-treated mice improved treatment efficacy (Panebianco et al., 2018; Routy et al., 2018). Recently, a clinical benefit was reported in a phase I trial with anti-PD-1 refractory metastatic melanoma patients who underwent FMT from donors with complete response after anti-PD-1 monotherapy (Baruch et al., 2020). In another clinical trial comparing outcomes in patients with metastatic hormone receptor positive BC who received eribulin (antitubulin antimitotic agent) with or without pembrolizumab (anti-PD-1 agent), a shift in the abundance of *Akkermansia* and *Faecalibacterium* after two cycles of therapy was described. However, alterations were only reported in patients receiving eribulin (Barroso-Sousa et al., 2020). These findings require future confirmation in larger cohorts and further studies in BC are needed to validate microbiota as a predictor of treatment efficacy.

Similarly to other anticancer treatments that induce immunogenic cell death, radiotherapy promotes systemic inflammation (Martina, 2020). Gut microbiota has been demonstrated to have a role in radiotherapy efficacy and toxicity (Eslami-S et al., 2020; Martina, 2020). Radiotherapy outcomes are highly dependent on tumour microenvironment (Poff et al., 2013; Martina, 2020). In contrast with normal tissue, tumour microenvironment may involve abnormal metabolism, including hypoxia, acidosis and necrosis (Duong et al., 2019). Low oxygen concentration in hypoxic tumour areas can be attractive for obligate anaerobes (e.g., *Clostridium* and *Bifidobacterium*) and facultative anaerobes (Duong et al., 2019). In fact, hypoxia cancer cells are three times more resistant to radiotherapy than well-oxygenated cells (Poff et al., 2013). Two promising treatments involve the use of hyperbaric oxygen therapy and nimorazole (antimicrobial with activity against anaerobic bacteria and protozoa) to reverse the cancer-promoting effects of tumour hypoxia (Thomson et al., 2014; Bennett et al., 2018). Furthermore, radiotherapy affects gut microbiota composition with a potential effect on vitamin D metabolism. Alteration of serum level of vitamin D may influence radiotherapy response through mechanisms associated with gut microenvironment, including immune cell response, bacterial metabolites and signalling pathways related to vitamin D receptors. Loss of DNA repair protein 53BP1 results in radioresistance of BC cells. Interestingly, the active form of vitamin D (25-hydroxyvitamin D3) stabilises 53BP1 levels in tumour cells, resulting in increased genomic instability in response to radiation and reduced cell proliferation (Huang et al., 2019). Gut microbiota is a double-edged sword for radiation response, with effects that can be either beneficial and protective or detrimental and resistant. *Lactobacillus sakei*, *L. acidophilus*, *L. casei*, and *Bifidobacterium* spp. are known to have a radioprotective effect (Huang et al., 2019). In contrast, there are ionising-radiation-resistant bacteria such as *Rubrobacter radiotolerans*, *Deinococcus radiodurans* R1, *R. xylanophilus*, *Chroococcidiopsis* spp., *Hymenobacter actinosclerus*, *Kineococcus radiotolerans*, *A. radioresistens*, *Kocuria rosea*, and *M. radiotolerans* (Cox and Battista, 2005). As described earlier, *M. radiotolerans* (D10-value of 1,000 Grey) and *A. radioresistens* (D10-value of 2,000 Grey) were found to be relatively abundant in oestrogen positive BC (including in sentinel lymph node) and postmenopausal BC patients, respectively (Xuan et al., 2014; Yazdi et al., 2016; Zhu et al., 2018). *Deinococcus radiodurans* is an extremely resilient radioresistant bacteria (D10-value of 10,000 Grey) that limits DNA damage through efficient mechanisms of proteins protection against oxidative stress and DNA repair, enhanced by functional redundancies in both systems (Cox and Battista, 2005; Slade and Radman, 2011). In the future, further investigation of what mechanisms make bacteria resistant to radiation could help improve response to treatments.

Nowadays, despite new modern radiotherapy techniques, delayed radiation injury can appear with a latency period of a few months to several decades (Bennett et al., 2016). Recently, a unique gut microbiota signature was identified in an animal model of late radiation-tissue injury, capable of increasing expression of IL-1β, IL-6, and TNF-α. Radiation significantly reduced the prevalence of Firmicutes and increased abundance of *Akkermansia*, *Bacteroides*, *Parabacteroides*, *Sutterella*, *Turicibacter*, and of an unclassified genus belonging to RF32 order (Gerassy-Vainberg et al., 2018).

In addition, new treatment combinations were investigated to explore the effect of probiotics on local BC tissue. However, it appears that breast microbiota cannot be modified even with long-term exposure to locally administered probiotics and therefore, the beneficial effects will be more related to gut microbiota modulation (Eslami-S et al., 2020).

These pharmacomicrobiomics studies may support the potential use of gut microbiota analysis to predict patients’ response to treatments, allowing a more personalised approach based on the microbiota-host-cancer triad. Our expectancies are that, in the near future, in-depth knowledge of interactions between therapeutic agents and microbiota will have a growing interest and impact clinical practice shifting treatment paradigms.

#### Potential Limitations and Future Perspectives

Since the advent of the latest next-generation sequencing (NGS) techniques, such as 16S rRNA and shotgun sequencing, the understanding of microbiota/microbiome and disease has been growing at an accelerated rate with promising results. These new laboratory methods allow deciphering microbiota at species-level, with high precision, detecting even small but significant changes.

However, there are still some limitations and biases that need to be considered. First, there are several confounding variables which can influence final interpretation, due to the fact that microbiota is modulated by a wide range of external factors, such as age, sex, body mass index, type of diet, and geographical location. In most studies, these confounding variables are not measured or balanced, and the number of patients is considered small and unrepresentative, which makes it difficult to extrapolate the results. Thus, drawing definitive conclusions of these data is risky, since microbiota has high heterogeneity between individuals and even within the same individual (O’Malley and Skillings, 2018).

Another relevant concern is methodological and technical comparisons between studies since there are different NGS methods to analyse microbiota (e.g., 16S rRNA and shotgun metagenome analysis). In some studies, there is a difference at the genus-level, while in others this variation is found at a species-level, increasing the difficulty of making inferences and extrapolations. In addition, statistical and bioinformatics analysis is not the same across the studies. Sample collection, preservation, transportation and time to analysis also impact results and are not, unfortunately, standardised.

With regards to the struggle for comparison between different studies, there are “top-down” researches, which means that microbiota is analysed as an ecosystem and, thus, dysbiosis is described as taxonomic/functional group changes, while that in “bottom-up” researches the focus is on specific microbial aspects and their mechanisms, regardless of the community effect. Based on this, the comparison between these two types of studies is fallacious and should be done with caution (O’Malley and Skillings, 2018).

Moreover, many of these studies are performed *in vitro*, which makes it difficult to conjecture the same assumptions to a living organism and to prove causality. Hence, to formulate inferences of causality, animal studies need to be carried out with a standardised methodology, such as the following: a group of gnotobiotic mice (e.g., germ-free mice) is transplanted with faeces from humans with a certain disease, and another group is transplanted with faecal material of healthy human participants. Then, a comparison is made between the two groups, evaluating signs and symptoms in the group of patients, in order to find altered mechanisms and dysbiosis.

However, the major drawback remains the fact that the microbiota is highly variable among individuals with a particular disease and even in the patient itself, including during the circadian rhythm. Furthermore, the recipient and the donor diverge in genetic, anatomical, physiological, and behavioural components, which may affect the reliability of causal inference. Therefore, this method may not reflect the real alterations in microbiota after transplant for gnotobiotic mice (Walter et al., 2020).

We propose some recommendations and future perspectives that may be considered in upcoming studies that intend to study the association of BC with the microbiota, allowing to overcome some of the limitations aforementioned:

• Design studies based on assumptions validated in previous studies (if existing) and related to the main objectives and the study setting (e.g., to determine the difference of the F/B ratio in patients with BC and a control group of healthy volunteers or comparison between gut microbiota profile between locally advanced BC and *de novo* metastatic BC patients).
• Stratification of the patients by different subgroups (e.g., postmenopausal state, omnivorous vs. vegan diet).
• To define precise inclusion and exclusion criteria in order to control confounding variables as much as possible (e.g., exclude patients with 3 months prior use of antibiotics, probiotics, prebiotics, symbiotics, laxatives, proton pump inhibitors, or Chinese herbal medicine).
• Control the conditions of collection, storage, and transport of biological samples (e.g., faecal samples stored at −80°C or, whenever possible, the collection should be done at the same timepoint of the day, in order to control individual circadian variations).
• Generally, bioinformatic analysis should be standardised, with the intention of validating comparison between studies. Some research groups have proposed methods for microbiota standardisation analysis (Lahti and Shetty, 2017).
• When performing microbiota studies in animal models, there is a need to transplant faecal material, either from healthy humans (part of the control group) and humans with a specific disease (part of the experimental groups) to gnotobiotic mice. Hence, swith the aim to evaluate whether the modifications of microbiota observed in human faeces are reflected in the animal model, the research should evaluate microbiota before and after the transplant. Furthermore, animal models, more similar to humans, as primates and pigs, should be used in order to produce more validated inferences.
• Whenever possible, with the intention to prove reproducibility and replicability, and to understand if the effect of microbiota modifications is, in fact, biological or the product of local/community influences, studies should be performed intercontinentally.
• In relation to the interplay between microbiota and the BC, it is suggested that the studies take into consideration, not only the bacterial segment of microbiota but also the virome (e.g., viral portion of microbiota), mycobiome (e.g., fungal portion of microbiota), and metabolomics (chemical processes concerning metabolites), in order to assess all the effects and relationships between each member of the respective microbial ecosystem.
• There is an unmet need to explore, which consists of possible mechanisms of gut microbiota modulation through prebiotics, probiotics, symbiotics, antibiotics, or personalised nutrition and, ultimately, even by faecal microbiota transplant.
• The capacity to understand the association between gut microbiota and breast microbiota is still lacking on how communication between them occurs, how they influence each other, whether the intestinal microbiota reflects the breast microbiota or vice versa and whether, modulating the gut microbiota, there is an echo in the breast microbiota (breast-gut axis).
• Finally, there is still a need to comprehend what are the processes that can cause, in some patients, what is called “cancer brain” or “chemo brain.” Possibly, exploring the back-and-forth of the gut-brain axis, BC metabolomics and pharmacomicrobiomics, a clearer and complementary explanation can be reached (Bajic et al., 2018; Ma et al., 2019).

Although the microbiota/microbiome is a rapidly evolving field and of transversal interest to science, there are still profound grey areas around the real links between BC and the millions of microbes that are part of the cellular and genetic heritage of its human host.

## Conclusion

Microbiota, the “unknown organ,” seems to have a remarkable impact on our organism. However, the links between BC and microbiota are complex and not yet fully understood. Gut and/or breast dysbiosis may contribute or even correspond to a precursor and facilitator state of breast oncogenic process.

With the use of multi-omics, an astonishing growing biotechnological area, we believe that it will be feasible, in the short-term, to demonstrate more precise links between BC and microbiota and to bring these findings into the clinical practice. Stronger and accurate translational and clinical studies are needed to support this rationale and speed access of patients to a new, fascinating and even more tailored therapeutic avenues.

In the near future, microbiota could be considered a non-hereditary risk factor for BC, potentially modulated by a personalised preventive and therapeutic approach. Likewise, in established BC, the microbiota may be a prognostic and predictive factor of response to treatment and/or its side effects. Also, modulation of microbiota can be used to improve outcomes in BC patients.

## Ethics Statement

This manuscript does not contain any studies with human participants or animals performed by any of the authors.

## Author Contributions

The present manuscript is the result of original work by the authors. DAC and JN: conception and design, development of methodology, acquisition, analysis, and interpretation of data. DAC, JN, MB, CR, CC, AC, MM, JC, and LC: writing, review, and/or revision of the manuscript. IN, PB, MB, SB, JC, and LC: manuscript supervision. All authors contributed to the article and approved the submitted version.

## Conflict of Interest

The authors declare that the research was conducted in the absence of any commercial or financial relationships that could be construed as a potential conflict of interest.
