# Supplementary material for: Human Microbiota and Breast Cancer—Is There Any Relevant Link?—A Literature Review and New Horizons Toward Personalised Medicine
Source: Front Microbiol. 2021 Feb 25;12:584332. doi: 10.3389/fmicb.2021.584332 (PMC7947609; doi:10.3389/fmicb.2021.584332)
Supplement: Supplementary file 1 [file Table_1.docx]

# **Supplementary Material**

**Influence of gut microbiota in anticancer therapies (including in breast cancer).**

| **Chemotherapy** | Anthracyclines | | | | |
| --- | --- | --- | --- | --- | --- |
|  | **Doxorubicin** | Pharmacokinetics | *Raoultellaplanticola* | *R. planticola*: doxorubicin deglycosylation into 7-deoxydoxorubicinol 7-deoxydoxorubicinolone | 68 |
|  |  |  | *Escherichia coli BW25113*  *Klebsiella pneumoniae* | Doxorubicin degradation dependent on molybdopterin-dependent enzymes |  |
|  |  | Toxic effects | *Raoultellaplanticola* | 🡻cardiotoxicity and other side-effects |  |
|  | Alkylating agents | | | | |
|  | **Cyclophosphamide** | Pharmacokinetics | *Enterococcus hirae*  *Lactobacillus johnsonii*  *Lactobacillus murinus* | **🡹** gut barrier permeability | 64 69 70 |
|  |  |  |  | **🡹**bacteria translocation to lymphoid organs |  |
|  |  |  |  | **🡹**Th1 and Th17 cells |  |
|  |  |  |  | **🡹** level of IFN-γ and IL-17 |  |
|  |  |  |  | **🡹** antitumour immunological response |  |
|  |  | Toxic effects | No studies available | | |
|  | Taxanes | | | | |
|  | **Paclitaxel** | Pharmacokinetics | No studies available* | | |
|  |  | Toxic effects | *Akkermansiamuciniphila* | 🡻 systemic inflammation and taxanes-induced neuropathic pain. | 71 |
|  |  |  | *Eubacterium siraeum*  *Lactobacillus intestinalis* | Inhibitors of pain phenotype |  |
|  | **Docetaxel** | Pharmacokinetics | No studies available* | | |
|  |  | Toxic effects | *Bifidobacterium breve* strain Yakult  *Lactobacillus casei* strain Shirota  Galactooligosaccharides | 🡻 severity: diarrhoea, lymphopenia and febrile neutropenia | 64  72 |
|  | Deoxycytidine analogues | | | | |
|  | **Gemcitabine** | Pharmacokinetics | Gammaproteobacteria | 🡻efficacy gemcitabine 🡪 Inactive form (nucleoside analogue-catabolising enzymes) | 64  73 |
|  |  | Toxic Effects | *Mycoplasma* spp. |  |  |
|  | Pyrimidine analogues | | | | |
|  | **5-fluorouracil**  **Capecitabine** | Pharmacokinetics | *Mycoplasma hyorhinis* | 🡻cytostatic activity (FdUrd and F3(d)Thd)  **🡹** cytostatic activity (5′dFUrd) | 64  76  77 |
|  |  | Toxic effects |  | 🡻toxicityafter FMT from healthy mice |  |
|  | Antimitotic agents | | | | |
|  | **Eribulin** | Pharmacokinetics | *Akkermansia*  *Faecalibacterium* | Shift in abundance after 2 cycles | 92 |
|  |  | Toxic effects |  |  |  |

| **Anti - HER 2** | HER2 inhibitors | | | | | |
| --- | --- | --- | --- | --- | --- | --- |
|  | **Trastuzumab** | Pharmacokinetics | Clostridiales | **🡹**efficacy (including 🡹pCR) | | 80 |
|  |  |  | *Lactococcus lactis*  *Lactobacillus paracasei* |  |  |  |
|  |  |  | Bacteroidales | 🡻efficacy | |  |
|  |  | Toxic effects | No studies available* | | | |
|  | **Pertuzumab** | Pharmacokinetics | No studies available* | | | |
|  |  | Toxic effects |  | Rifaximin: 🡻 PIGO | | 81 |
|  | **Trastuzumab emtansine** | Pharmacokinetics | No studies available* | | | |
|  |  | Toxic effects | No studies available* | | | |
|  | **Neratinib** | Pharmacokinetics | No studies available* | | | |
|  |  | Toxic effects | No studies available* | | | |
|  | **Tucatinib** | Pharmacokinetics | No studies available* | | | |
|  |  | Toxic effects | No studies available* | | | |
| **Hormonal therapy** | Selective oestrogen modulators/degraders | | | | | |
|  | **Tamoxifen** | Pharmacokinetics |  | 🡻 BC risk: Tamoxifen + Daidzein | | 83 |
|  |  |  |  | 🡻 BC risk: Tamoxifen + Genistein | |  |
|  |  | Toxic effects | No studies available* | | | |
|  | **Raloxifen** | Pharmacokinetics | No studies available* | | | |
|  |  | Toxic effects | No studies available* | | | |
|  | **Fulvestrant** | Pharmacokinetics | No studies available* | | | |
|  |  | Toxic effects | No studies available* | | | |
|  | Aromatase inhibitors | | | | | |
|  | **Letrozole** | Pharmacokinetics |  | 🡻Bacteroidales  **🡹**🡻Firmicutes | **🡹**increasedadiposity | 82 |
|  |  | Toxic effects | No studies available* | | | |
|  | **Anastrozole** | Pharmacokinetics | No studies available* | | | |
|  |  | Toxic effects | No studies available* | | | |
|  | **Exemestane**  **+/- everolimus (mTOR kinase inhibitor)** | Pharmacokinetics | No studies available* | | | |
|  |  | Toxic effects | No studies available* | | | |
|  | GnRH agonists | | | | | |
|  | **Goserelin** | Pharmacokinetics | No studies available* | | | |
|  |  | Toxic effects | No studies available* | | | |
|  | **Leuprorelin** | Pharmacokinetics | No studies available* | | | |
|  |  | Toxic effects | No studies available* | | | |
|  | **Triptorelin** | Pharmacokinetics | No studies available* | | | |
|  |  | Toxic effects | No studies available* | | | |

| **Newer drugs** | Cyclin inhibitors | | | | | | |
| --- | --- | --- | --- | --- | --- | --- | --- |
|  |  | Pharmacokinetics | No studies available* | | | | |
|  |  | Toxic effects | No studies available* | | | | |
|  | PARP inhibitors | | | | | | |
|  |  | Pharmacokinetics | No studies available* | | | | |
|  |  | Toxic effects | No studies available* | | | | |
| **Immunotherapy** | Anti-PD-1 | | | | | | |
|  | **Nivolumab**  **Pembrolizumab** | Pharmacokinetics | **🡹***Bacteroidescaccae*  **🡹***Bifidobacteriumlongem*  **🡹***Collinsellaaerofaciens*  **🡹***Enterococcusfaecium*  **🡹***Faecalibacteriumprausnitzii*  **🡹***Lachnospiraceae*  **🡹***Veillonellaceae* | **🡹**systemic and antitumour immunity | | | 64  80  88  89  90  91 |
|  |  |  | **🡹***Ruminococcaceae* | **🡹**efficacy with FMT from responders | | |  |
|  |  |  | **🡹**Microbiota diversity |  |  |  |  |
|  |  | Toxic effects | No studies available* | | | | |
|  | Anti-PD-L1 | | | | | | |
|  |  | Pharmacokinetics | *Akkermansiamuciniphila*  *Bifidobacterium* | **🡹**T cell response | | | 80  89  90  91 |
|  |  | Toxic effects | No studies available* | | | | |
|  | Anti-CTLA-4 | | | | | | |
|  | **Ipilimumab** | Pharmacokinetics | *Bacteroidescaccae*  *Bacteroidesfragilis*  *Bacteroidesthetaiotaomicron* | **🡹**efficacy by Th1 immune response | | | 64  80  85  86 |
|  |  | Toxic effects | *Bacteroidaceae*  *Barnesiellaceae*  *Rikenellaceae* | 🡻 levels of bacteria | | | 64 85 |
|  |  |  |  | 🡻 polyamine transport | | |  |
|  |  |  |  | 🡻 vitamin B biosynthesis | | |  |
|  |  |  |  | **🡹**risk of colitis | | |  |
| **Radiotherapy** | Radiotherapy | | | | | | |
|  |  | Pharmacokinetics | *Rubrobacterradiotolerans* | D10 value | 11,000 Gy | Radioresistant effect | 98 |
|  |  |  | *Deinococcusradiodurans* R1 |  | 10,000 Gy |  |  |
|  |  |  | *Rubrobacterxylanophilus* |  | 5,500 Gy |  |  |
|  |  |  | *Chroococcidiopsis*spp. |  | 4,000 Gy |  |  |
|  |  |  | *Hymenobacteractinosclerus* |  | 3,500 Gy |  |  |
|  |  |  | *Kineococcusradiotolerans* |  | 2,000 Gy |  |  |
|  |  |  | *Acinetobacter radioresistens* |  | 2,000 Gy |  |  |
|  |  |  | *Kocuria rosea* |  | 2,000 Gy |  |  |
|  |  |  | *Methylobacteriumradiotolerans* |  | 1,000 Gy |  |  |
|  |  | Toxic effects | *Bifidobacterium* spp.  *Lactobacillus sakei*  *Lactobacillus acidophilus*  *Lactobacillus casei* | Radioprotective effect | | | 97 |
|  |  |  | 🡻 Firmicutes  **🡹***Akkermansia*  **🡹***Bacteroides*  **🡹***Parabacteroides*  **🡹***Sutterella*  **🡹***Turicibacter*  **🡹**genus RF32 order | Late radiation-tissue injury  **🡹**IL-1β, IL-6 and TNF-α | | | 101 |

BC (breast cancer); CTLA-4 (cytotoxic T-lymphocyte–associated antigen 4); D10 value (dose needed to eradicate 90% of irradiated population); F3(d)Thd (5-trifluorothymidine); FdUrd (5-fluoro-2'-deoxyuridine); 5-fluoro-5ʹ-deoxyuridine (5′dFUrd); FMT (faecal microbiota transplant); GnRH (gonadotropin-releasing hormone); Gy (Gray); HER2 (human epidermal growth factor 2); IFN-γ (interferon-gamma); IL (interleukin); mTOR (mammalian target of rapamycin); PARP (poly-ADP ribose polymerase); pCR (pathological complete response); PD-L1 (programmed death-ligand 1); PIGO (pertuzumab-induced gastrointestinal toxicity); Th (T helper); TNF-α (tumour necrosis factor-alpha). Peach colour (chemotherapy agents); green colour (HER2 inhibitors); yellow colour (hormonal therapy); purple colour (newer drugs); blue colour (immunotherapy); grey colour (radiotherapy); * To the best of our knowledge
